# Virus-Specific Differences in Rates of Disease during the 2010 Dengue Epidemic in Puerto Rico

**DOI:** 10.1371/journal.pntd.0002159

**Published:** 2013-04-04

**Authors:** Tyler M. Sharp, Elizabeth Hunsperger, Gilberto A. Santiago, Jorge L. Muñoz-Jordan, Luis M. Santiago, Aidsa Rivera, Rosa L. Rodríguez-Acosta, Lorenzo Gonzalez Feliciano, Harold S. Margolis, Kay M. Tomashek

**Affiliations:** 1 Epidemic Intelligence Service, Centers for Disease Control and Prevention, Atlanta, Georgia, United States of America; 2 Dengue Branch, Division of Vector-Borne Diseases, Centers for Disease Control and Prevention, San Juan, Puerto Rico; 3 Puerto Rico Department of Health, San Juan, Puerto Rico; Emory University, United States of America

## Abstract

**Background:**

Dengue is a potentially fatal acute febrile illness (AFI) caused by four mosquito-transmitted dengue viruses (DENV-1–4) that are endemic in Puerto Rico. In January 2010, the number of suspected dengue cases reported to the passive dengue surveillance system exceeded the epidemic threshold and an epidemic was declared soon after.

**Methodology/Principal Findings:**

To characterize the epidemic, surveillance and laboratory diagnostic data were compiled. A suspected case was a dengue-like AFI in a person reported by a health care provider with or without a specimen submitted for diagnostic testing. Laboratory-positive cases had: (i) DENV nucleic acid detected by reverse transcriptase-polymerase chain reaction (RT-PCR) in an acute serum specimen; (ii) anti-DENV IgM antibody detected by ELISA in any serum specimen; or (iii) DENV antigen or nucleic acid detected in an autopsy-tissue specimen. In 2010, a total of 26,766 suspected dengue cases (7.2 per 1,000 residents) were identified, of which 46.6% were laboratory-positive. Of 7,426 RT-PCR-positive specimens, DENV-1 (69.0%) and DENV-4 (23.6%) were detected more frequently than DENV-2 (7.3%) and DENV-3 (<0.1%). Nearly half (47.1%) of all laboratory-positive cases were adults, 49.7% had dengue with warning signs, 11.1% had severe dengue, and 40 died. Approximately 21% of cases were primary DENV infections, and 1–4 year olds were the only age group for which primary infection was more common than secondary. Individuals infected with DENV-1 were 4.2 (95% confidence interval [CI]: 1.7–9.8) and 4.0 (95% CI: 2.4–6.5) times more likely to have primary infection than those infected with DENV-2 or -4, respectively.

**Conclusions/Significance:**

This epidemic was long in duration and yielded the highest incidence of reported dengue cases and deaths since surveillance began in Puerto Rico in the late 1960's. This epidemic re-emphasizes the need for more effective primary prevention interventions to reduce the morbidity and mortality of dengue.

## Author Summary

Dengue is a potentially fatal acute febrile illness that is endemic throughout the tropics and sub-tropics. Dengue has been endemic in Puerto Rico for several decades and recent epidemics occurred in 1994–5, 1998 and 2007. In January 2010, dengue surveillance indicated that an epidemic had begun. The epidemic peaked in early August and ended in December with a total of 26,766 suspected dengue cases identified, of which 128 were fatal. The 2010 epidemic was one of the longest in Puerto Rico history and resulted in the greatest number of cases and deaths ever detected. We analyzed the epidemiologic and immunologic characteristics of laboratory-confirmed dengue cases and age group-specific attack rates, and determined the frequency of first DENV infection and DENV-types among persons experiencing their first infection. This analysis indicated that 10–19 year-olds were most affected during the epidemic, and that DENV-1 was roughly four times more likely to be associated with clinically apparent illness upon first DENV infection than were DENV-2 or -4. The 2010 dengue epidemic demonstrated the heavy burden of illness due to dengue in Puerto Rico, re-emphasizing the critical need for effective primary prevention tools to reduce the morbidity and mortality due to dengue worldwide.

## Introduction

Dengue virus (DENV) transmission is endemic throughout most of the tropics and sub-tropics and is estimated to result in ∼50 million symptomatic infections and ∼20,000 deaths each year [1], [2]. Infection with any DENV can result in dengue, an illness characterized by fever, headache, retro-orbital eye pain, myalgia and rash [2]. In some cases, dengue can progress to severe dengue [2], which includes dengue hemorrhagic fever (DHF) and dengue shock syndrome (DSS) [3] and is characterized by thrombocytopenia, increased vascular permeability with plasma leakage, severe organ involvement, and/or clinically significant bleeding [2]. Supportive care with appropriate intravascular volume repletion has been shown to lower mortality associated with severe dengue [2].

The four related but serotypically distinct DENV-types, DENV-1, -2, -3 and -4, are transmitted by *Aedes aegypti* or *Ae. albopictus* mosquitoes [4], [5]. Following infection, individuals develop short-lived, heterotypic immunity and long-lived, type-specific immunity [6]. Primary infection is an individual's first DENV infection, and secondary infection is any subsequent infection with a DENV-type different from the first. Severe dengue is more common upon secondary infection [2], [7] and may be affected by the order in which an individual is infected with the respective DENV-types [2], [8]. Thus, increases in the force of DENV infection can result in a decrease in the age of primary and secondary infection [2]. Both local patterns of circulation of the four DENV-types and force of infection can influence the age groups most affected by dengue and severe dengue.

The unincorporated United States territory of Puerto Rico is composed of 78 municipalities, an area of 3,515 square miles, and a population of 3,725,789 [9]. The demographics of Puerto Rico are similar to the United States as median age is 36 years and 78.6% are white, although 99% are self-described Hispanic [9]. Since the mid-1990's the health care system in Puerto Rico has included both public and private health care services, and dengue has been a reportable condition for several decades. *Ae. aegypti* is the predominant DENV vector on the island.

Dengue was first described in Puerto Rico in 1915 [10] and outbreaks have been recognized since 1963 [11], [12]. DHF was first reported in 1975 [13], [14], all four DENV-types have been identified on the island since 1982 [15], [16], and the first confirmed dengue-related death was reported in 1986 [17]. Recent epidemics were detected in 1994–1995, 1998 and 2007, with 24,700 [18], 17,000 [19] and 10,508 [20] reported suspect cases, respectively (Table S1). During both epidemic and non-epidemic periods, 10–19 year olds have been the most affected age group for several decades.

In the present investigation, we describe a dengue epidemic that occurred in 2010, including differences in the epidemiology of cases infected with different DENV-types with respect to primary versus secondary infection.

## Materials and Methods

### Investigation design

A retrospective analysis of suspected dengue cases reported to surveillance systems was performed to: 1) describe the epidemiology of the 2010 dengue epidemic, including disease severity; 2) determine the proportion of primary and secondary DENV infections, and the molecular epidemiology of the DENVs responsible for the epidemic; and 3) describe relationships between demographic variables (e.g. age, sex, municipality of residence) and characteristics of illness (e.g. infecting DENV-type, severity of illness). This investigation underwent institutional review at CDC and was determined to be public health practice and not research, including the post-hoc determinations of DENV molecular epidemiology and primary/secondary infection rates in reported cases; as such, Institutional Review Board approval was not required.

### Data sources

Surveillance data from five sources were used to identify cases. First, Centers for Disease Control and Prevention Dengue Branch (CDC-DB) and Puerto Rico Department of Health (PRDH) jointly operate the island-wide Passive Dengue Surveillance System (PDSS) that requires an acute serum specimen and completion of a Dengue Case Investigation Form (DCIF) (cdc.gov/dengue/resources/dengueCaseReports/DCIF_English.pdf) for case reporting and diagnostic testing. Second, the Enhanced Dengue Surveillance System (EDSS) operates solely in the municipalities of Patillas and Guayama and utilizes an on-site nurse epidemiologist to encourage case reporting and patient follow-up to obtain a convalescent serum specimen [21]. Third, identification of fatal dengue cases is conducted via PDSS and EDSS [22], and enhanced fatal case surveillance was initiated in January 2010 in collaboration with the Instituto de Ciencias Forenses de Puerto Rico, which obtains blood and tissue specimens at autopsy from suspected dengue-related deaths. Fourth, PRDH operates the Notifiable Diseases Surveillance System (NDSS) wherein suspected dengue cases are reported without diagnostic testing at CDC-DB. Last, in addition to dengue diagnostic testing performed at CDC-DB for PDSS and EDSS, testing is performed by two private diagnostic laboratories outside of Puerto Rico according to their internal protocols [23]. Diagnostic test results from these laboratories and patient data, including sex, age, and date of illness onset (if unavailable, specimen collection date was used instead), were entered into an independent database. Deduplication of individuals reported to more than one data source was achieved by matching records on name and date of birth and consolidation into a single case if two or more reports from any data source had symptom onset dates within 14 days of each other. As case-patients' travel history is not well captured via the surveillance systems used in this investigation, reported cases may represent both locally-acquired as well as travel-associated cases.

### Dengue diagnostic testing

All diagnostic testing was performed at CDC-DB for serum specimens received through PDSS or EDSS using the following algorithm: acute specimens (collected ≤5 days after symptom onset) were tested by DENV-type-specific real-time reverse-transcriptase-polymerase chain reaction (RT-PCR) [24] adapted for high throughput using MDX-10 Universal and M48 systems (Qiagen, Valencia, CA); acute specimens collected 5 days after symptom onset and all convalescent specimens (collected ≥6 days after symptom onset) were tested for the presence of anti-DENV immunoglobulin M (IgM) antibody with an antibody-capture enzyme-linked immunosorbent assay (MAC ELISA) and a cut-off value of the OD_450_ of the specimen versus that of the negative control (ie. P/N ratio ) ≥2.0 [25], [26]. All serum specimens from fatal cases were tested by both RT-PCR and MAC ELISA. Tissue specimens were tested at CDC Infectious Diseases Pathology Branch in Atlanta, GA by immunohistochemistry (IHC) [27] and flavivirus-specific RT-PCR [28] followed by sequencing.

### Definitions

A suspected dengue case was a dengue-like illness in a person in Puerto Rico whose health care provider: 1) submitted a DCIF and serum or tissue specimen to CDC-DB for dengue diagnostic testing; 2) submitted a serum specimen to a private laboratory for dengue diagnostic testing; or 3) reported the case via NDSS.

A laboratory-positive case was a suspected dengue case that met the following criteria for current (criteria 1 and 2) or recent (criterion 3) DENV infection: 1) detection of DENV nucleic acid in a serum or tissue specimen; 2) detection of DENV antigen in a tissue specimen; or 3) detection of anti-DENV IgM antibody in a serum specimen.

A laboratory-negative case was a suspected dengue case with: 1) no anti-DENV IgM antibody detected in a convalescent specimen; or 2) no DENV nucleic acid or antigen detected in a fatal case with only a tissue specimen submitted.

A laboratory-indeterminate case was a suspected dengue case with no DENV nucleic acid or anti-DENV IgM antibody detected in an acute specimen with no convalescent specimen available for testing.

Dengue with warning signs and severe dengue were defined according to 2009 WHO clinical guidelines [2]; dengue, DHF and DSS were defined according to 1997 WHO clinical guidelines [3].

### Primary and secondary DENV infections

A representative sample of all RT-PCR-positive cases reported to PDSS or EDSS with illness onset between January 1 and December 31, 2010 was selected to determine the rates of primary and secondary DENV infection. Cases were stratified by age group with optimal allocation to allow for comparison between age groups, and further allocated to reflect the proportion of DENV-types that occurred during 2010 to allow for comparison between DENV-types and age groups. Sample size was calculated using an estimate of the proportion of secondary infections by age group based on data from the 2007 dengue epidemic [20], an error of 20%, 95% significance, and an expected 20% of specimens having insufficient specimen volume remaining for testing to be completed. Of the 1,000 selected cases, 818 had sufficient specimen volume and were tested at a dilution of 1∶100 for the presence of anti-DENV IgG antibody by ELISA using DENV-1–4 antigen and a cut-off value of OD_450_≥0.15 [29], [30]. A secondary DENV infection was defined by detection of anti-DENV IgG antibody in an acute specimen, and a primary DENV infection by lack of anti-DENV IgG antibody detection in an acute specimen.

### Sequencing and phylogenetic analysis

Serum specimens with DENV-1 (n = 7), DENV-2 (n = 2) or DENV-4 (n = 4) detected by RT-PCR were randomly selected from municipalities with the highest incidence of the respective DENV-type and inoculated into cultured C6/36 cells; the presence of virus was confirmed by RT-PCR and indirect immunofluorescence [31]. Isolates were further propagated and viral RNA was extracted from culture supernatants using the M48 BioRobot System (Qiagen; Valencia, CA). The envelope glycoprotein (E) gene was amplified and sequenced; sequence data were restricted to the E gene open reading frame (1,485 basepairs). Multiple sequence alignment was performed using MUSCLE available in MEGA 5 (megasoftware.net) and GTR+Γ+I4 was selected as the best nucleotide substitution model as determined by MODELTEST v3.7. Genetic relatedness was inferred and represented with phylogenetic trees using the maximum likelihood method in MEGA 5. MCMC was run in BEAST v1.6.1 (beast.bio.ed.ac.uk) under Bayesian skyline prior, constructed in TreeAnnotator found in the same BEAST package, and visualized in FigTree v1.3. Both trees rendered almost identical tree topologies, therefore confirming genetic relatedness. Evolutionary distances were corroborated by pairwise alignment in BioEdit v7.1.3 and E gene sequences from GenBank were included in the phylogenetic tree to support tree topology by currently circulating genotype. Tree topology was supported by bootstrapping with 1,000 replicates. Genotypes were referred to by previously described nomenclature [32], [33].

### Statistical analyses

The frequencies of clinical, demographic and laboratory data were calculated by performing descriptive analyses of all suspected dengue cases identified in 2010. Rates of suspected dengue and laboratory-positive cases were calculated using population denominators obtained from the 2010 United States Census [9]. Statistical differences in proportions were tested by applying the Chi-squared test and Fisher's exact test when applicable. Unless otherwise noted, relative risk ratios were used to calculate all differences between effect sizes. All data analyses were conducted using SAS 9.2 (SAS Institute Inc., Cary, NC), graphs were produced in Microsoft Excel (Microsoft Corp., Redmond, WA), and maps were created using ArcView (ESRI, Redlands, CA).

## Results

### Suspected cases

**Figure 1:**
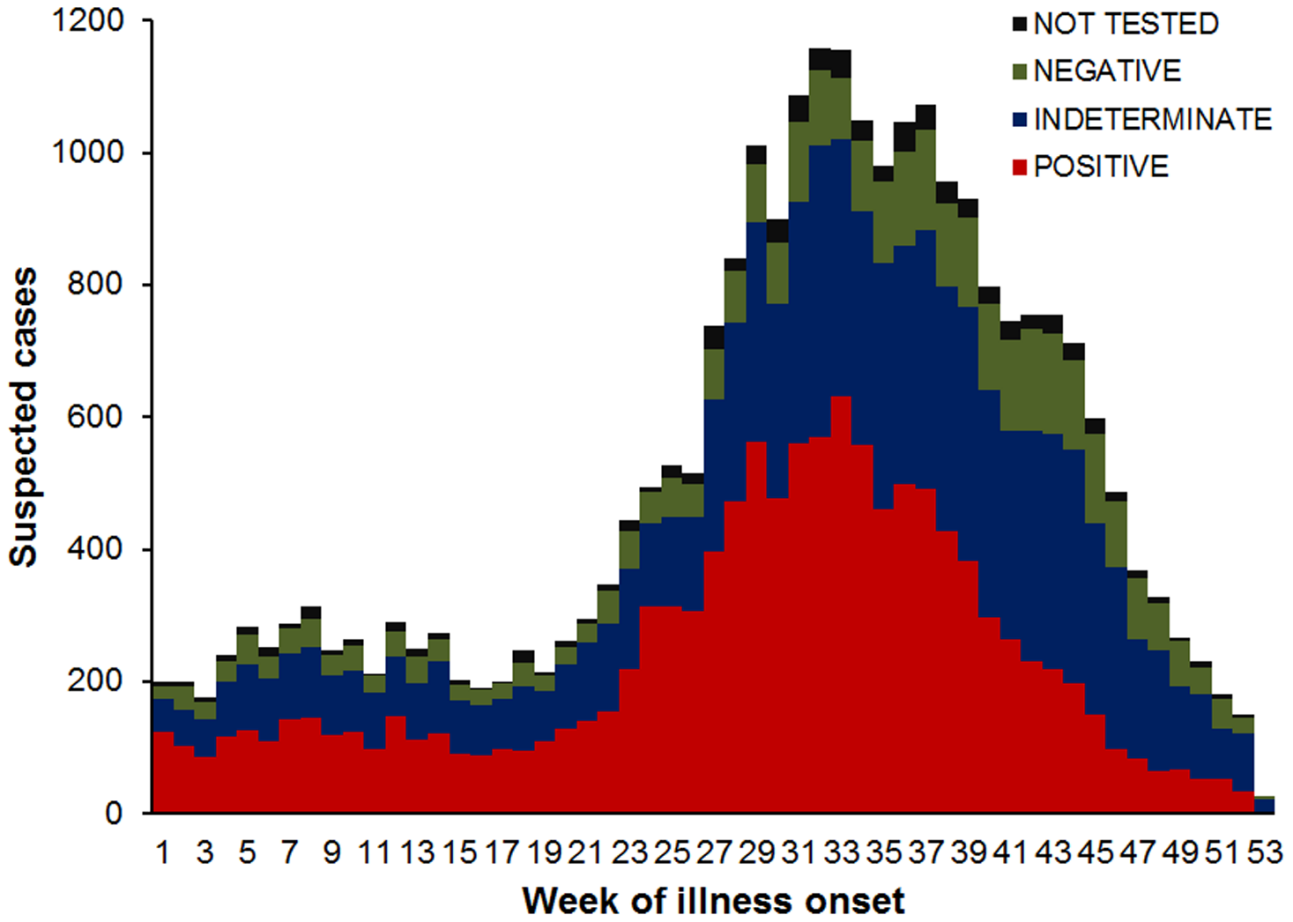
Epidemic curve of suspected dengue cases by week of illness onset, Puerto Rico, 2010. Surveillance data from cases reported via the Passive Dengue Surveillance System, Enhanced Dengue Surveillance System, Notifiable Diseases Surveillance System, or private laboratory dengue diagnostic test results were compiled and grouped by diagnostic test result as indicated.

We identified 26,766 suspected dengue cases with illness onset between January 1 and December 31, 2010 (7.2 suspected dengue cases per 1,000 residents). Of these, 22,496 (84.0%) were reported to PDSS, 1,846 (6.9%) were identified though diagnostic testing at a private laboratory, 1,304 (4.9%) were reported to NDSS, and 1,120 (4.2%) were reported to EDSS (Fig. S1). Suspected dengue cases exceeded the PDSS epidemic threshold in the first week of 2010, increased steeply in week 20 (May 14–20), and peaked at 1,157 in week 32 (August 6–12) (Fig. 1). Suspected dengue cases slowly declined thereafter and returned to below the historic average in mid-December.

Of all suspected dengue cases, 25,852 (96.6%) had a specimen tested for evidence of DENV infection, of which 25,246 (97.7%) were tested by CDC-DB and the remainder by a private laboratory; paired specimens were available for 1,996 (7.5%) cases. Of all cases with a specimen tested, 3,664 (14.2%) were laboratory-negative, 10,140 (39.2%) were laboratory-indeterminate, and 12,048 (46.6%) were laboratory-positive (3.2 laboratory-positive cases per 1,000 residents). The median weekly proportion of cases that tested laboratory-positive was 48.3%, and was highest (64.5%) in week 24 (June 11–17) and lowest (11.1%) in week 53 (December 31).

### Laboratory-positive cases

**Figure 2:**
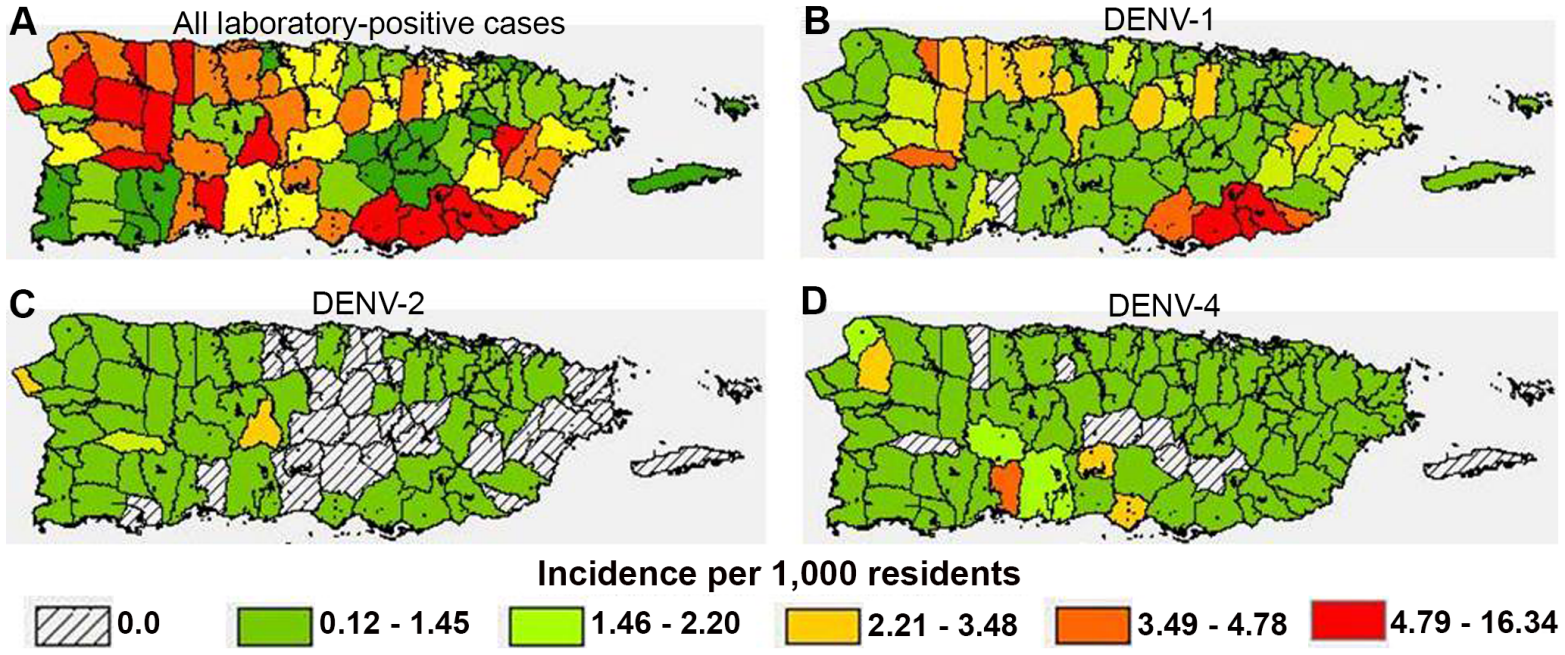
Rates of laboratory-positive cases by municipality, Puerto Rico, 2010. Rates were calculated by dividing case numbers by municipality-specific populations and grouping by quintile of rate of all laboratory-positive cases. Rates shown are: (**A**) All laboratory-positive cases; or laboratory-positive cases with DENV-1 (**B**), DENV-2 (**C**), or DENV-4 (**D**) detected by RT-PCR.

Laboratory-positive case-patients resided in all 78 municipalities of Puerto Rico (Fig. 2A), and the median rate of laboratory-positive cases by municipality was 2.68 per 1,000 residents. Rates were the highest in the municipality of Patillas (16.34 cases per 1,000 residents), the southeastern municipality where the EDSS site is located [21], and lowest in Aibonito (0.12 cases per 1,000 residents) in the mountainous center of Puerto Rico. Of 7,426 RT-PCR-positive cases, DENV-1 was detected in 5,126 (69.0%) and incidence was highest in the southeast (Fig. 2B). DENV-2 was detected in 545 (7.3%) cases primarily in the west (Fig. 2C), whereas DENV-4 was detected in 1,757 (23.7%) cases and incidence was highest in south-central and northwestern Puerto Rico (Fig. 2D). DENV-3 was detected in just two (<0.1%) cases in early 2010.

**Figure 3:**
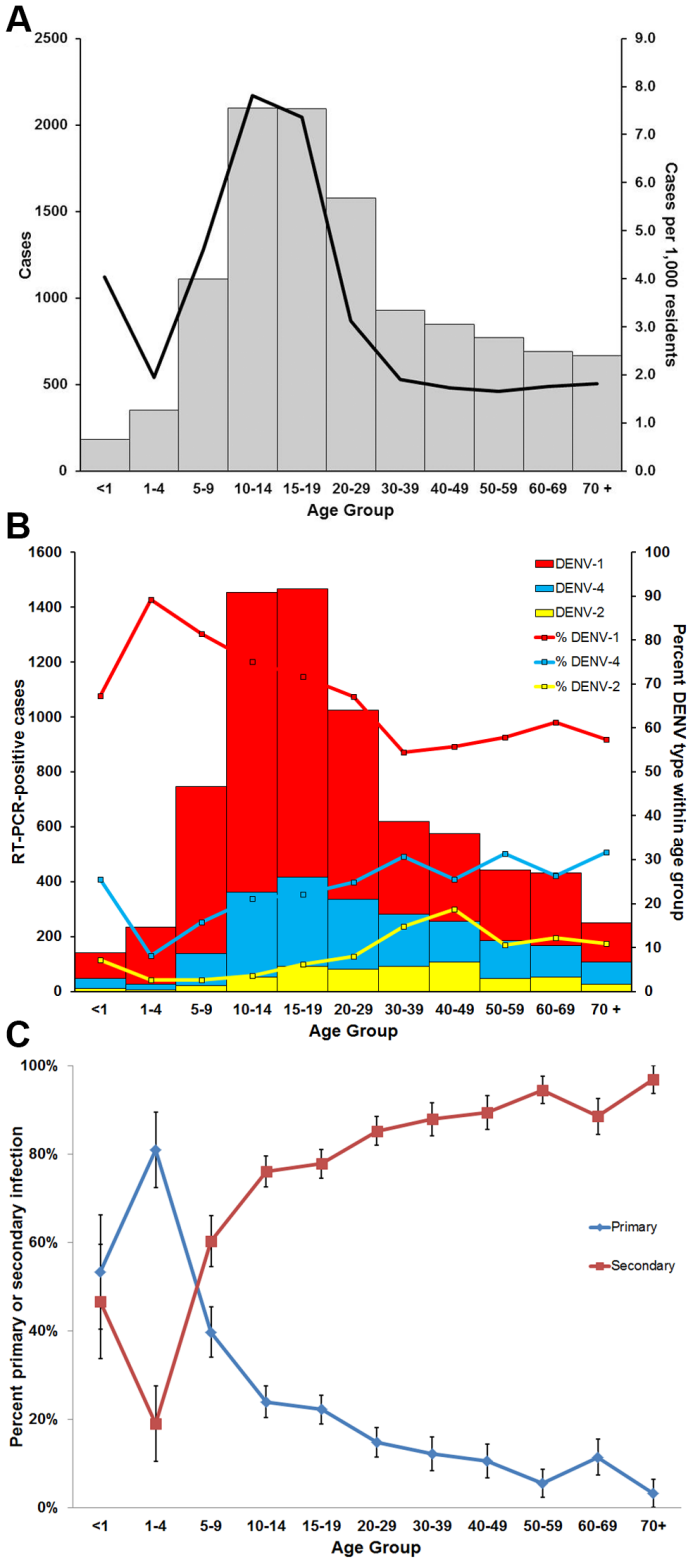
Age distribution of laboratory-positive cases, Puerto Rico, 2010. **A**: Age distribution and rates of laboratory-positive cases; **B**: Age distribution and incidence of RT-PCR-positive cases by infecting DENV-type; **C**: Primary and secondary DENV infections by age group from a representative sample of RT-PCR-positive cases; error bars indicate standard error of the mean; denominators by age group are 15, 21, 73, 146, 162, 115, 74, 66, 54, 61 and 31, respectively.

The age distribution of laboratory-positive cases was significantly different from suspected dengue cases only for case-patients between 30 and 69 years of age (Fisher's exact, p≤0.04). The median age of laboratory-positive case-patients was 18 years (Table 1). The most affected age group was 10–14 year olds (7.8 cases per 1,000 individuals), followed by 15–19 year olds (7.4 cases per 1,000 individuals) (Fig. 3A). Five-to-nine year olds were the next most affected age group followed by individuals <1 year of age (4.6 and 4.1 cases per 1,000 individuals, respectively). Individuals 50–59 years of age were the least affected age group (1.7 cases per 1,000 individuals).

**Table 1:** Demographic and clinical characteristics of suspected dengue cases by diagnostic test result, Puerto Rico, 2010.

	Suspected	Laboratory-positive	Laboratory-negative	Laboratory-indeterminate
	(N = 26,766 )	(N = 12,048)	(N = 3,664)	(N = 10,140)
Male, n (%)	14,332 (53.5)	6,628 (55.0)	1,858 (50.7)	5,364 (52.9)
Median age, years (range)	18 (5 days–102 years)	18 (1 month–102 years)	21 (1 week–90 years)	17 (5 days–100 years)
Hemorrhagic manifestation, n (%)	7,031 (26.3)	3,805 (31.5)	756 (20.6)	2,470 (24.4)
Dengue, n (%)	17,126 (64.0)	8,911 (74.0)	1,757 (48.0)	6,458 (63.7)
Dengue with warning signs, n (%)	10,836 (40.5)	5,991 (49.7)	1,100 (30.0)	3,745 (36.9)
Severe dengue, n (%)	2,680 (10.0)	1,334 (11.1)	393 (10.7)	953 (9.4)
DHF, n (%)	448 (1.7)	289 (2.4)	60 (1.6)	99 (1.0)
Death, n (%)	128 (0.5)	40 (0.3)	64 (1.7)	24 (0.2)

DHF = dengue hemorrhagic fever.

The distribution of RT-PCR-positives cases among age groups was not significantly different from that of laboratory-positive cases (Fisher's exact, p>0.05) except for the 50–59 year-old age group, for which serum specimens were collected later (median: 6 days post-illness onset [DPO]) than all other age groups (median: 4 DPO) (Fisher's exact, p = 0.04) and thus tested less frequently by RT-PCR. Despite this, the distribution of DENV-types was not consistent among age groups (Fig. 3B). The strong majority (89.3%) of RT-PCR-positive cases in individuals 1–4 years of age were due to infection with DENV-1, whereas 8.1% and 2.6% were due to infection with DENV-4 and -2, respectively. The percent of infections due to DENV-1 decreased and those due to DENV-4 increased with age until a plateau of approximately 60% DENV-1, 30% DENV-4 and 10% DENV-2 was reached in the 20–29 year old age group.

### Primary and secondary DENV infections

From the sample of 818 RT-PCR-positive specimens tested for primary versus secondary DENV infection, 169 (20.7%) were primary and 649 (79.3%) were secondary. The median age of individuals experiencing primary infection was 14 years, compared to 23 years for individuals experiencing secondary infection. Eighty-one percent of individuals 1–4 years of age had primary infection and were the only age group for which primary infection was significantly more common than secondary (p = 0.003) (Figure 3C). More than 89% of infections in all adult age groups (i.e. age ≥20 years) were secondary. The frequency with which anti-DENV IgG antibody was detected in specimens taken from infants was likely due to the presence of maternal antibody [2].

Whereas 28.5% of all DENV-1 infections were primary, significantly fewer DENV-2 (6.8%) and DENV-4 (7.1%) cases were primary infections (p<0.0001) (Table 2). Calculation of relative risk ratios (RR) indicated that individuals infected with DENV-1 were 4.2 and 4.0 times more likely to be experiencing primary infection than were individuals infected with DENV-2 or -4, respectively (Table 2).

**Table 2:** Clinical characteristics of laboratory-positive cases by infecting dengue virus (DENV)-type, Puerto Rico, 2010.

	DENV-1	DENV-2	DENV-4	DENV-1 vs. DENV-2		DENV-1 vs. DENV-4		DENV-2 vs. DENV-4	
	N = 5,126	N = 545	N = 1,757						
	n (%)	n (%)	n (%)	RR	95% CI	RR	95% CI	RR	95% CI
Primary infection*	148 (28.5)	5 (6.8)	16 (7.5)	**4.2**	**1.7–9.8**	**4.0**	**2.4–6.5**	1.0	0.4–2.5
Hemorrhagic manifestation	1,537 (30.0)	150 (27.5)	518 (29.5)	1.1	0.9–1.3	1.0	0.9–1.1	0.9	0.8–1.1
Dengue	4,151 (81.0)	442 (81.1)	1,457 (82.9)	1.0	1.0–1.0	1.0	1.0–1.0	1.0	0.9–1.0
Dengue with warning signs	2,717 (53.0)	275 (50.5)	956 (54.4)	1.1	1.0–1.1	1.0	0.9–1.0	0.9	0.8–1.0
DHF	93 (1.8)	17 (3.1)	77 (4.4)	0.6	0.3–1.0	**0.4**	**0.3–0.6**	0.7	0.4–1.2
Severe dengue	434 (8.5)	55 (10.1)	250 (14.2)	0.8	0.6–1.1	**0.6**	**0.5–0.7**	**0.7**	**0.5–0.9**
Fatal dengue	21 (0.41)	5 (0.91)	10 (0.57)	0.4	0.2–1.2	0.7	0.3–1.5	1.6	0.6–4.7

Relative risk ratios (RR) were calculated with 95% confidence intervals (CI) for the indicated outcomes for case-patients infected with DENV-1, DENV-2, or DENV-4. Bolded data indicate a significant risk in the indicated outcome associated with infection with the indicated DENV-type. DHF = dengue hemorrhagic fever.

* based on a sample of 818 RT-PCR-positive specimens that were tested for evidence of primary infection; denominators for DENV-1, -2 and -4 are 520, 73, and 225, respectively.

### Molecular epidemiology

**Figure 4:**
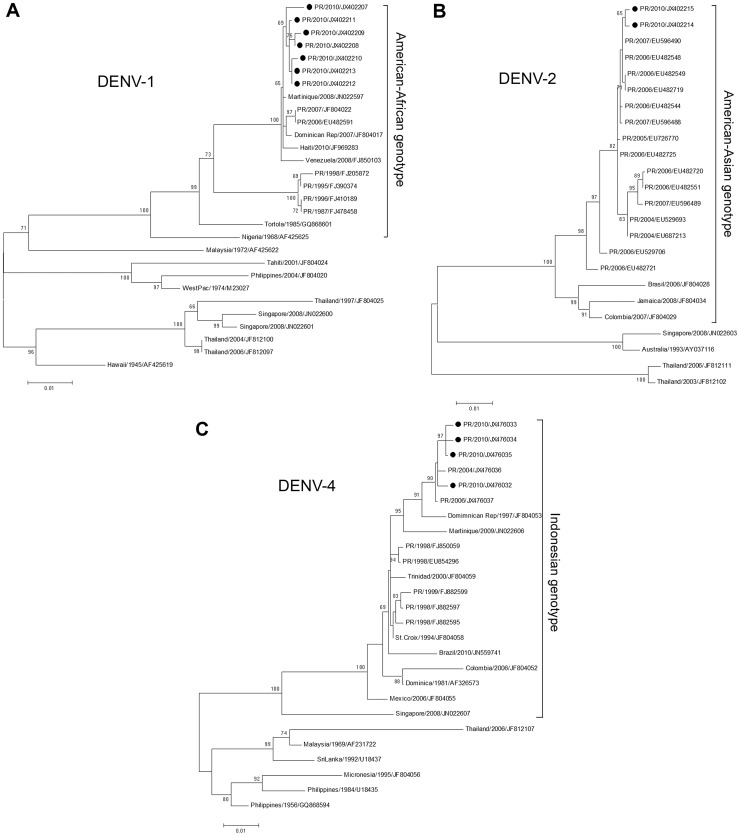
Maximum likelihood trees depicting the phylogenetic relationships of DENV-1, -2, and -4 isolated in Puerto Rico, 2010. Each phylogeny was tested with 1,000 bootstrapping cycles. Each taxa label consists of country of origin (PR = Puerto Rico), year of virus isolation, and GenBank accession number. Viruses isolated and sequenced for this investigation are labeled with a black dot. Genotype names were based on previously published phylogenies [32], [33]. All outgroups have been removed. **A**: Phylogenies were constructed using 29 DENV-1 E gene sequences: seven from Puerto Rico in 2010, and 22 obtained from GenBank to represent the three main genotypes: American-African, South Pacific, and Asian. **B**: Phylogenies were constructed using 24 DENV-2 E gene sequences: two from Puerto Rico in 2010, and 22 obtained from GenBank to represent the three main genotypes: American-Asian, Cosmopolitan, and Asian II. **C**: Phylogenies were constructed using 26 DENV-4 E gene sequences: four from Puerto Rico in 2010, and 22 obtained from GenBank to represent the two main genotypes: Indonesian and South East Asian.

Sequencing and phylogenetic analyses of randomly selected DENV isolates showed that DENV-1 belonged to the American-African genotype (genotype V [34]), but to a clade distinct from virus isolated during the 1998 Puerto Rico epidemic (Fig. 4A). Available sequence data suggest that close ascendants of the 2010 DENV-1 clade had been circulating in Puerto Rico and the Caribbean since at least 2006 (Fig. 4A). DENV-2 sequencing indicated that the virus belongs to clade 1B of the American-Asian genotype (genotype IIIb [35]) (Fig. 4B), which is composed of DENV strains endemic to Puerto Rico [36]. DENV-4 belonged to the Indonesian genotype (genotype II [37]), but was distinct from virus isolated in 1998 (Fig. 4C). Viruses closely-related to the DENV-4 isolated in 2010 were first detected in Puerto Rico in 2004 (Fig. 4C).

### Disease severity

Of 12,048 laboratory-positive cases, 31.5% had at least one hemorrhagic manifestation and sufficient clinical data was provided to classify 74.0% as dengue and 2.4% as DHF (Table 1). Nearly half (49.7%) of all laboratory-positive cases had dengue with at least one warning sign, and 11.1% had severe dengue. Of 128 suspected dengue deaths, 40 (31.3%) were laboratory-positive cases. While adults represented nearly half of laboratory-positive cases with dengue (47.1%), dengue with warning signs (44.6%), and severe dengue (49.7%), they accounted for nearly all (92.5%) fatal dengue cases. Laboratory-positive severe and fatal dengue occurred at a rate of 0.36 and 0.01 cases per 1,000 residents, respectively; laboratory-positive fatal dengue cases occurred at a rate of 30.0 per 1,000 severe dengue cases. From the sample of cases for which primary and secondary DENV infection status was determined, secondary infection was identified in 102 (87.9%) case-patients with severe dengue and 547 (77.9%) case-patients without severe dengue (RR = 1.2; 95% CI = 1.1–1.2).

Case-patients with DHF were more likely to have been infected with DENV-4 than DENV-1, and those with severe dengue were more likely to have been infected with DENV-4 than DENV-1 or -2 (Table 2). There was no significant difference between infection with DENV-1, -2 or -4 and likelihood of being a fatal case.

## Discussion

In 2010, Puerto Rico experienced the largest and longest dengue epidemic ever documented on the island. In total, more than 12,000 individuals had laboratory-confirmed dengue, of which more than 1,300 experienced severe dengue and 40 died. The most common DENV identified was DENV-1, and 1–4 years old were the only age group more frequently experiencing a primary versus secondary DENV infection. Individuals infected with DENV-1 were four times more likely to have a primary infection than were those infected with DENV-2 or -4. A strength of this investigation was utilization of multiple surveillance systems to identify all reported suspect dengue cases. However, a minor weakness was that data obtained from each system may not be directly comparable due to different diagnostic algorithms used by CDC-DB and private laboratories, and we were not able to determine status of primary versus secondary infection or perform sequencing on specimens from private laboratories. Because private laboratories contributed <5% of all laboratory-positive dengue cases, this likely did not affect the conclusions of this investigation.

The 2010 dengue epidemic was similar in several respects to the 1998 epidemic: both began in January during El Niño events accompanied by above average temperatures, which while not a determinant of epidemics in Puerto Rico [38] may contribute to increased DENV transmission [39]; and both epidemics peaked in week 32 of the calendar year and were predominated by transmission of DENV-1 and -4 [19]. A notable difference was that DENV-3 was essentially absent in 2010, whereas it accounted for ∼6% of cases during the 1998 epidemic [19]. DENV-3 was re-introduced into Puerto Rico in 1998 following a 20-year absence and was the predominant virus-type in the 2007 dengue epidemic [20]. Thus, susceptibility to DENV-3 infection was likely high in 1998 and low in 2010, which likely explains these observations.

The American-African and Indonesian genotypes of DENV-1 and -4 have been circulating in Puerto Rico since introduced in 1978 and 1981, respectively [16], [40]. However, the DENV-1 isolated in 2010 was distinct from the DENV-1 isolated during the 1998 epidemic (Fig. 4A and [41]) and was more closely related to the DENV-1 isolated during the 2007 epidemic (Fig. 4A). Similarly, the DENV-4 isolated during the 2010 epidemic was distinct from the DENV-4 isolated in 1998 and was more closely related to viruses circulating since 2004 (Fig. 4B). These findings suggest that DENV-1 and -4 may have both experienced clade replacements at some point after 1998 but prior to 2007. After the re-introduction of DENV-3 into Puerto Rico in 1998, DENV-1 was not detected between 2001 and 2006 and DENV-4 was not detected between 2000 and 2005 [42]. Nonetheless, apparent re-introductions of DENV-1 in 2007 and DENV-4 in 2006 were soon followed by the disappearance of DENV-3 in 2010 (this paper and [42]). In place of the convenience sample used in this investigation to describe the DENVs responsible for the epidemic, sequencing of a representative sample of specimens and longitudinal sequence analysis will be necessary to both confirm apparent clade replacements and determine if other DENV clades contributed to the 2010 epidemic.

Similar to previous epidemics in Puerto Rico (Table S1), 10–19 year olds were most affected during the 2010 epidemic; however, unlike previous epidemics, 5–9 year olds were the next most affected age group. The median age of individuals experiencing secondary DENV infection declined from 27 years in 2007 [20] to 23 years in 2010, likely due to the relative proximity of the periods of high infection pressure. Taken together, these observations indicate an increase in incidence of dengue and a decrease in the age of secondary infection, suggesting that the overall force of DENV transmission may have been higher in 2010 than in previous epidemic years.

The observation that DENV-2 and -4 cause relatively infrequent clinical apparent illness upon primary DENV infection is consistent with previous studies [43]–[48]. Similarly, our finding that DENV-1 was a more frequent cause of clinically apparent illness upon primary infection has also been previously reported [43], [49], including the observation of increased disease severity during primary infection with DENV-1 compared to other DENV-types [44], [50], [51]. Nonetheless, of 545 DENV-2 and 1,755 DENV-4 infections, roughly 7% were primary, indicating that primary infection with these DENVs can cause clinically apparent illness, contrary to previous assertions [46], [47]. The relative abundance of DENV-1 compared to DENV-2 and -4 is unlikely to be responsible for the observed differences in likelihood of causing clinically apparent illness upon primary infection, as relative risk ratios compare the proportion of exposed individuals experiencing the outcome of interest. This is supported by the findings in the 1–4 year-old age group, of which ∼80% experienced a primary infection with DENV-1. Alternative explanations for these observations include potential variations in the sensitivity of detection of DENV-type-specific anti-DENV IgG antibody and differences in force of infection between the DENV-types circulating in 2010.

We also saw that DENV-1 and -2 were less frequently a cause of severe dengue than DENV-4. This is in contrast to previous studies where DENV-1 was a more frequent cause of DHF than DENV-4 [52], and a study where DENV-2 was twice as likely to result in DHF as DENV-4 [43]. Possible explanations for these differences include: the comparatively small number of DENV-4 infections observed in previous studies; differences in clade and/or viral fitness leading to differential pathogenicity [33], [53], [54]; and/or the DENV-type(s) and sequence to which individuals were previously exposed, which may affect the likelihood of developing severe dengue [44], [55], [56].

This investigation had several limitations. First, because individuals experiencing secondary infection may have a diminished anti-DENV IgM antibody response [57], suspected dengue cases tested solely for anti-DENV IgM antibody may have been misclassified. Second, although DENV is the sole flavivirus known to cause clinically apparent illness in humans in Puerto Rico (CDC, unpublished data), some proportion of anti-DENV IgM or IgG positive results could have been due to infection with or vaccination against another flavivirus [58], resulting in misclassification. Third, because clinical data was provided for >90% of case-patients on only one occasion and some data variables were incompletely reported (e.g. only 56% of suspected cases had a reported status of hospitalization), severity of disease and the rates of dengue with warning signs and severe dengue reported here were likely underestimated. Finally, the description of the epidemiology and molecular characteristics of dengue reported here is only representative of reported, clinically apparent DENV infections and may not be reflective of asymptomatic and sub-clinical DENV infections.

The 2010 dengue epidemic in Puerto Rico demonstrated that dengue continues to be a public health concern for Puerto Rico residents and visitors, and surveillance systems and control initiatives should continue to be supported and strengthened. This epidemic also highlights the need for effective primary prevention tools such as a dengue vaccine to reduce disease morbidity and mortality.

## Supporting Information

Table S1 Summary of epidemiologic data from previous dengue epidemics in Puerto Rico.
(DOCX)

Figure S1 Flow diagram of data sources, diagnostic test results, and sub-analyses of suspected dengue cases, Puerto Rico, 2010. **A**: Data sources and diagnostic test results. **B**: Sub-analyses using RT-PCR-positive specimens. PDSS = Passive Dengue Surveillance System; PrivLab = private diagnostic laboratories; NDSS = National Disease Surveillance System; EDSS = Enhanced Dengue Surveillance System; IHC = immunohistochemistry; IgG ELISA = anti-DENV immunoglobulin G enzyme-linked immunosorbent assay; RT-PCR = real-time reverse-transcriptase polymerase chain reaction; DENV = dengue virus; * = includes two co-infections.
(TIF)
