# Supplementary material for: Virus-Specific Differences in Rates of Disease during the 2010 Dengue Epidemic in Puerto Rico
Source: PLoS Negl Trop Dis. 2013 Apr 4;7(4):e2159. doi: 10.1371/journal.pntd.0002159 (PMC3617145; doi:10.1371/journal.pntd.0002159)
Supplement: Table S1 — Summary of epidemiologic data from previous dengue epidemics in Puerto Rico. (DOCX) [file pntd.0002159.s001.docx]

**SUPPORTING TABLE 1**

| **Epidemic Year** | **DENV-type(s)*** | **Reported suspect cases**  (cases per  1,000 population) | **Most affected age groups**^*^ | **Reported DHF cases**  (DHF cases per  1,000 dengue cases) | **Reported dengue deaths**  (deaths per  1,000 dengue cases) | **Citation** |
| --- | --- | --- | --- | --- | --- | --- |
| 1963 | DENV-3 | 27,000^ (NA) | NA | 0 (0) | NA | [[11](#_ENREF_11)] |
| 1969 | DENV-2 | 16,665 (NA) | NA | 0 (0) | 0 (0) | [[12](#_ENREF_12)] |
| 1977 | DENV-2, -3, -1 | 12,733 (3.75) | 15–19, 20–29, 10–14 | 0 (0) | 0 (0) | [[14](#_ENREF_14)] |
| 1986 | DENV-4, -1, -2 | 10,659 (NA) | 6–15, 31–45, <1 | NA | 3^†^ (0.3) | [[17](#_ENREF_17)] |
| 1994-5 | DENV-2, -4, -1 | 24,700 (7.0) | 15–19, 10–14, 20-24 | 152 (6.2) | 40 (1.6) | [[18](#_ENREF_18)] |
| 1998 | DENV-4, -1, -2, -3 | 17,000 (4.8) | 10-19, <1 | 174 (10.2) | 56 (3.3) | [[19](#_ENREF_19)] |
| 2007 | DENV-3, -2, -1, -4 | 10,508 (2.7) | 10–14, 15–19, <1 | 227 (21.6) | 40 (3.8) | [[20](#_ENREF_20)] |
| 2010 | DENV-1, -4, -2, -3 | 26,766 (7.2) | 10–14, 15–19, 5-9 | 448 (16.7) | 128 (4.8) | This manuscript |

* = in order of relative frequency

^ = estimated number of cases

^†^ = laboratory-positive deaths; number of suspected deaths not available

DENV = dengue virus

DHF = dengue hemorrhagic fever

NA = data not available
