# Supplementary figures and images for: Virus-Specific Differences in Rates of Disease during the 2010 Dengue Epidemic in Puerto Rico
Source: PLoS Negl Trop Dis. 2013 Apr 4;7(4):e2159. doi: 10.1371/journal.pntd.0002159 (PMC3617145; doi:10.1371/journal.pntd.0002159)

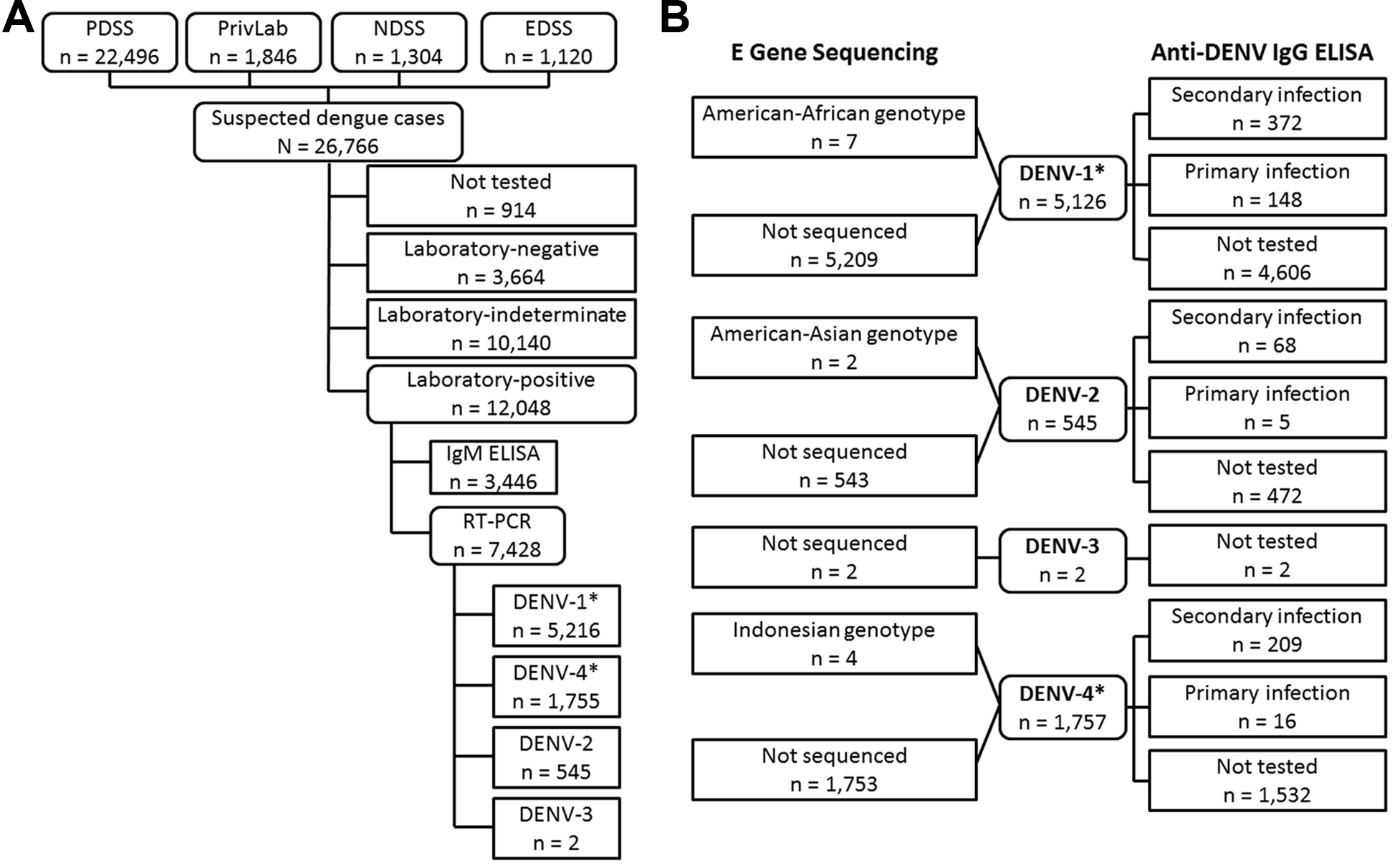

Supplement: Figure S1 — Flow diagram of data sources, diagnostic test results, and sub-analyses of suspected dengue cases, Puerto Rico, 2010. A: Data sources and diagnostic test results. B: Sub-analyses using RT-PCR-positive specimens. PDSS = Passive Dengue Surveillance System; PrivLab = private diagnostic laboratories; NDSS = National Disease Surveillance System; EDSS = Enhanced Dengue Surveillance System; IHC = immunohistochemistry; IgG ELISA = anti-DENV immunoglobulin G enzyme-linked immunosorbent assay; RT-PCR = real-time reverse-transcriptase polymerase chain reaction; DENV = dengue virus; * = includes two co-infections. (TIF) [file pntd.0002159.s002.tif]
